# Addressing the global surge of COVID-19 cases: Insights from diagnostics, improved treatment strategies, vaccine development and application

**Published:** 2021-03-12

**Authors:** Kamoru A. Adedokun, Ayodeji O. Olarinmoye, Lawal O. Olayemi, Muhammed R. Shehu, Jelili O. Mustapha, Ramat T. Kamorudeen, Sulaimon A. Nassar

**Affiliations:** ^1^Department of Oral Pathology, DUH, King Saud University Medical City, Riyadh, Saudi Arabia; ^2^Department of Agriculture and Industrial Technology, Babcock University, Ilisan Remo, Ogun State, Nigeria; ^3^School of Medicine, Faculty of Health Sciences, National University of Samoa, Samoa; ^4^Department of Environmental Science, Southern Illinois University, Edwardsville, Illinois USA; ^5^Molecular Diagnostics Unit, DynaLIFE Medical Labs, Edmonton, Alberta, Canada; ^6^Department of Public Health, University of South Wales, Pontypridd, UK; ^7^Children Welfare Unit, Osun State Hospital Management Board, Asubiaro, Osogbo, Osun State, Nigeria; ^8^Department of Medical Laboratory Sciences, College of Health Sciences, Ladoke Akintola University, Ogbomoso, Oyo State, Nigeria

**Keywords:** antiviral drug, containment operation, COVID-19 vaccines, diagnostics, immunotherapy, SARS-CoV-2

## Abstract

**Background and aim:**

As the rage of coronavirus disease 2019 (COVID-19) pandemic continues to spread globally, much effort is being directed to contain it through various efforts – genomic studies, drug discoveries, clinical trials, vaccine development, and the innovation of diagnostic techniques. However, some pertinent areas involving accurate and sensitive diagnostics, immunoglobulin specificity, evolution of mutant strains of severe acute respiratory syndrome coronavirus 2 (SARS-CoV-2), and the drug combination strategy to combat it still require more attention.

**Methods:**

This review critically examines the COVID-19 response and containment operations. It also addresses some standing challenges involving the areas of diagnostics, vaccine development and prospect, and treatment strategies in relation to antiviral drug treatment and immunotherapy. Designated set of keywords such as “SARS-CoV-2;” “coronavirus;” “severe acute respiratory syndrome coronavirus;” “repurposed;” “vaccination;” “containment;” “laboratory diagnostic;” “immunotherapy;” “antiviral;” “antiparasitic;” “antibiotic;” “antiprotozoal;” “antibody;” “anti-inflammatory;” “antitumor;” “corticosteroid;” “hypertensive drug;” “statin;” “supplement;” and “biological” along with “COVID-19” were inserted on electronic databases to retrieve articles and clinical trial information relevant to the study objectives. The search databases included ClinicalTrials.gov, NIH.gov, PubMed, Scinapse, CINAHL, Medline, Google Scholar, Academic Search Premier, SAGE, EBSCO Host, and Scopus.

**Relevance for patients:**

The difficulties associated with SARS-CoV-2 rapid mutations are unceasingly evolving and re-evolving. These pose serious concerns and downplay the efficacy and effectiveness of the current pipeline antiviral drugs and vaccines. Entities encompassing immunotherapy, antiviral drug therapies, viral genomics, protein-protein interaction, and improved diagnostics as well as drug combination strategy against the emerging genetic variability of SARS-CoV-2 were critically appraised. This study suggests that robust collaborations in the development of more sensitive, rapid and accurate diagnostics, development of immunoglobulin specific agents and improved anti-viral treatment focus against multiple mutant genes of SARS-CoV-2 should be aggressively pursued for the overall benefits of COVID-19 patients.

## 1. Introduction

The year 2019 ended with a global alarm in the form of an outbreak of a new strain of coronavirus called the Severe Acute Respiratory Syndrome Coronavirus Type 2 (SARS-CoV-2). On December 31, 2019, the World Health Organization (WHO) alerted the global health community of the occurrence of a cluster of pneumonia cases in Wuhan city in the Hubei Province of China [1]. The toll of COVID-19 in terms of morbidity and mortality has been massive, especially in countries such as the United States, Italy, Spain, Germany, France, Republic of Korea and Iran [2]. As COVID-19 continues to spread worldwide, much effort is being directed by governments, the WHO, and other organizations to contain it through travel restrictions and social distancing. Researchers have swung into various actions including genomic studies, clinical trials, vaccine development, drug discoveries, and the innovation and improvement of diagnostic techniques. However, these efforts have resulted in limited success as the disease continues to spread unabatedly.

Recently, a big concern erupted over the dynamic antigenic drift of SARS-CoV-2 due to the discovery of about 14 mutant strains. From the available report, the existing immunogens and diagnostic reagents are normal products of the sequence of spike proteins from the index strain from Wuhan [3]. By indication, alteration in these spike proteins, the most studied domain in SARS-CoV-2 could result in reduced sensitivity to neutralizing antibodies and false testing outcomes against any new strains. Sooner or later, the emergence of new strains may outpace the ongoing interventions including vaccine design, diagnostic development, and the efficacy of the drug treatments.

Many therapeutic agents have also been tested against SARS-CoV-2, so far, very few remain hopeful. Even though there is currently no specific drug available to treat COVID-19, a number of clinical trials are premised on repurposed drugs, biologicals, and occasionally dietary supplementations. These therapeutic agents are believed to serve various curative and preventive purposes either alone or in combination. Major classes among these medications are: Antivirals (such as remdesivir, lopinavir/ritonavir, favipiravir, darunavir, ribavirin, elbasvir, tegobuvir, nelfinavir, and arbidol); broad spectrum anti-parasitics (such as nitazoxanide); broad spectrum synthetic serine protease inhibitor (such as nafamostat); antimalarials (such as artemisinin/artesunate, chloroquine, and hydroxychloroquine); antibiotics (such as azithromycin); antibodies/biologicals (such as interferon b1b and tocilizumab); and antitumor drugs (such as ruxolitinib), among others [4-6], Table 1 [7-30]. Sadly, the ongoing emergency choices in repurposed drugs are not without associated problems, mainly, adverse drug reactions that have resulted in cardiac problems, liver and gastrointestinal system disorders in those that are treated [31-34]. Lopinavir-ritonavir and interferon-b combination have been associated with self-limited nausea and diarrhea [35]. Chloroquine/Hydroxychloroquine therapy at therapeutic doses has been associated with side effects including electrocardiographic changes, retinopathy, myopathy, headaches, dizziness, and gastrointestinal upset [6]. Myopathy and rhabdomyolysis are the most frequent adverse effects of Statins [36]. Darunavir is associated with increased risk of myocardial infarction, especially in HIV co-infected patients [37], while Ribavirin plus Lopinavir/Ritonavir combination caused a significant increase in adverse gastrointestinal effects [38]. The common adverse effects of Remdesivir in severely infected COVID-19 patients have included nausea, acute respiratory failure, increased alanine aminotransferase (ALT) and constipation [39], and liver toxicity linked to tocilizumab [6]. However, there is currently a dearth of information to determine whether some of these adverse events are a result of the unstable SARS-CoV-2 genetic components, or due to individual patient response which may demand new treatment strategies.

**Table 1:** Selected repurposed therapeutic agents in COVID-19 clinical trials and possible adverse effects

Therapeutic class	Repurposed agents/intervention	Stage/expected completion date	Mode of action	Possible adverse effects	Trial registration	Locations	References
Anti-virals	Remdesivir	Phase III/II/March 2022	Inhibits viral RNA-dependent polymerase and induces a negative proofreading activity (Figure 2).	Respiratory failure and organ impairment, anaphylactoid reactions, heart rhythm disorders angioedema, diarrhea, skin rash, and hypotension, among others.	NCT04575064	Germany	[7,8]
	Lopinavir/Ritonavir	Phase III/March, 2021	Lopinavir is a peptidomimetic protease inhibitor combined with ritonavir. (Figure 2). Targets the main protease (Mpro) of SARS-CoV-2, a key protease enzyme required for the virus to replicate and assemble itself.	Allergic reaction, erectile dysfunction, libido, arrhythmia, abdominal pain, hyperlipidemia, nausea, among many others.	NCT04364022	Switzerland	[8,9]
	Favipiravir	Phase III/September, 2020	Inhibits the viral RNA synthesis through RNA-dependent RNA polymerase (RdRP, RDR) inhibition.	Impaired erythropoiesis. High concerns for gestational problems such as embryonic premature death and teratogenicity or congenital consequences.	NCT04425460	China	[8,10]
Anti-malarials	Artemisinin/Artesunate	Phase II/April, 2021	Possesses anti-inflammatory properties, such as inhibition of IL-6 that plays a key role in the development of severe COVID-19 and cytokine storm. Furthermore, inhibits NF-kB and viral protein synthesis, thus disrupting the viral replication process at early phase.	Possible parasite mutation may bolster drug-resistant strains (especially, in areas with endemicity of Plasmodium falciparum). Also, possibility of hemolytic anemia, severe allergic reactions and kidney failure.	NCT04387240	Saudi Arabia	[11-14]
	Chloroquine phosphate	Phase January 1, 2021	Inhibits glycosylation of the cellular ACE-2 receptor thereby interferes with binding of virus to the cell receptor. Furthermore, increases endosomal pH, thus interfering with fusion of SARS-CoV-2 and the host cell membranes	Cardiac problems. Retinopathy. Possibility of bone marrow suppression. Hypoglycemia, rash, and nausea.	NCT04443270	Mexico	[15-17]
	Hydroxychloroquine tablet	Phase IV/March, 2025	Increases endosomal pH, and interferes with SARS-CoV-2 binding. Inhibition of cytokine storm.	Headache, retinopathy, dizziness, gastrointestinal upset, myopathy, hypoglycemia, irregular heartbeat, rash, and nausea.	NCT04316377	Europe	[16,18]
Antibiotics	Azithromycin	Phase III/September, 2020	Possesses antiviral properties by preventing virus entry into cells, and replication. Enhances immune response against the virus, by upregulating the syntheses of type I and III interferons – particularly interferon-b and interferon-l. Antibacterial properties by inhibiting bacterial protein synthesis, thus preventing secondary infection.	Risk of bacterial resistance, hearing impairments, and pulmonary problems, on the long-term use.	NCT04381962	United Kingdom	[19-22]
Antitumor	Bevacizumab	Phase II/Completed	Binds circulating VEGF and blocks its receptor binding, effectively inhibiting downstream signaling and preventing angiogenesis, lymphangiogenesis, and potential edematous effect. VEGF is upregulated in COVID-19, and thus may contribute to pulmonary edema, leading to ARDS and ALI.	Possibilities of increased risk of infection, high blood pressure, peripheral neuropathy, nosebleed, rectal bleeding, headache, back pain, dry/watery eyes, dry/flaky skin, runny nose, sneezing, and changes in sense of taste.	NCT04275414	China	[23,24]
Others	Sarilumab	Phase II/III/Completed	A human monoclonal antibody binds to IL-6 receptors that inhibit IL-6-mediated signaling (IL-6 antagonist). In COVID-19, the IL-6 cytokine is plays a vital role in the inflammatory process and response in body system.	There are possibilities for anaphylactoid reactions, rash, urticaria, gastrointestinal perforation and new primary malignancy. There is a high accidental drug-drug interaction with several medications and vaccines. Serious concern for possible deadly infections. Active TB, invasive fungal, bacterial, viral, and other opportunistic infections have been experienced in patients receiving sarilumab.	NCT04315298	United States	[25,26]
Natural products	Omega-3, Nigella Sativa, Indian Costus, Quinine, Anise Seed, Deglycyrrhizinated Licorice, Artemisinin, Febrifugine	Phase II/III/December 4, 2020	Clinical immunity boosting to effective antiviral effect. Omeg-3 as an example affects the human health by many mechanisms, for example, antioxidant, immunity-boosting agent. Moreover, Omega-3 exerts an antiviral effect on Flu virus by inhibiting influenza virus replication 1. On the other hand, black seed supplementation exerts a chelation effect on sickle cell anemia patients and inhibits Human Heme Metabolism 2. Moreover, black seed exerts an antiviral effect on the replication of old coronavirus and the expression of (TRP-genes) family 3. In addition, Omega-3 regulates the human immunity against bacterial and viral infections	-	NCT04553705	Saudi Arabia	[27]
Dietary supplement	Antioxidant formulation (reduced GSH, NAC, SOD and bovine lactoferrin and immunoglobulin)	Not Applicable/April, 2021	Reactive oxygen species induce oxidative stress responses and thereby provoke acute lung injury (clinical feature of COVID-19 disease). Thus, antioxidant measure is expected to ameliorate or prevent the effect of oxidative damage. NAC can change the redox balance towards reduced status inside neutrophils by GSH, which suppresses NF-kB activation at concentrations of 10 mM or more, resulting in modulation of cytokine production and chemotactic signals	Gastrointestinal disturbances and diarrhea are possible. In addition, NAC is known to cause bleeding disorder and has high probability of drug interactions with some medications.	NCT04466657	Nigeria	[28-30]

ACE-2: Angiotensin-converting enzyme 2; ARD: Acute respiratory distress syndrome; ALI: Acute lung injury; GSH: Reduced glutathione; IL-6: Interleulin-6; NAC: N-acetylcysteine; NF-kB: Nuclear Factor kappa-light-chain-enhancer of activated B cells; SOD: Superoxide dismutase; VEGF: Vascular endothelial-derived growth factor

Furthermore, studies into the development of COVID-19 vaccines are underway with some vaccine trials showing promising outcomes. It is important to note that the emergence of SARS-CoV-2 rapid mutations may prompt various challenges against the success of the ongoing vaccines, including potential immunopathological reactions based on the knowledge of vaccine development from MERS and SARS [40,41]. It is also worthy of note that vaccines for MERS and SARS diseases are yet to be approved. Apart from the concerns over the success of SARS-CoV-2 vaccine design, the poor diagnostics that currently pose a huge threat to COVID-19 containment operations are another source of worry [42]. A study report has it that due to poor sensitivity and low reliability, several available antibody test kits have failed to detect early (pre-symptomatic) infection of COVID-19 and failed to identify asymptomatic carriers, who constitute the largest proportion of COVID-19 patients [42].

The present report, therefore, examines and addresses some current, budding and future challenges, which include diagnostics, vaccine development and prospect, and treatment strategies in relation to antiviral therapy. It also offers some useful research information to the body of available data, which may facilitate the containment and response operations against the global battle of COVID-19.

## 2. Evidence of Zoonotic Transmission and Transmission Pathways of SARS-CoV-2 Virus

**Figure 1:**
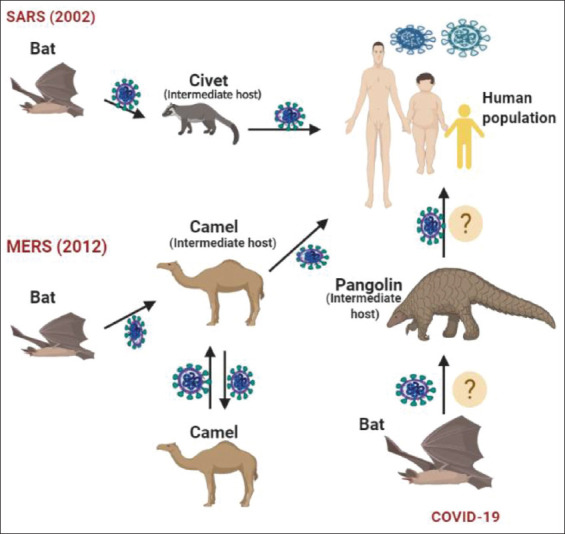
Potential transmission pathways of SARS-CoV-2 and similar coronavirus outbreaks

Familial and nosocomial contacts and community spread fuel and sustain the current rapid human-human transmission of SARS-CoV-2 virus in many countries [43]. Despite the rapid spread of the virus, there has been very little interest in identifying how the index case originally occurred, compared with the effort and resources being expended in search of cure and prevention. Nonetheless, the source of contact is widely believed to be a wet market in Wuhan where wild animals are traded [44]. in addition, it has been established that the first SARS virus (SARS-CoV) was initially transmitted from bats to civet cats and in MERS-CoV, from bats to camels before transmission to humans became possible in their transmission pathways. However, the potential reservoir and intermediate hosts of SARS-CoV-2 have not been conclusively identified (Figure 1).

The SARS-CoV-2 virus is a Beta-coronavirus, same as MERS-CoV and SARS-CoV; all the three viruses have their origins in bats. However, in the case of SARS-CoV-2, it is pangolins that have somewhat cautiously been implicated as the intermediate hosts [45,46]. Recent evidence-based phylogenetic comparison of SARS-CoV-2 with other closely related reference genomes revealed up to 96% similarity with bat SARS-like coronavirus strain, BatCov RaTG13 [47]. In addition, the detection of SARS-CoV-2 in human airway epithelial cell lines (Vero E6 and Huh-7) and transgenic animals show characteristic induction of multifocal pneumonia and interstitial hyperplasia [47]. These findings suggest that SARS-CoV-2 invades the pulmonary and intestinal tracts. Although, there is no definite agent identified with the index case till date, available phylogenetic evidence in combination with the cellular tropism shows that SARS-CoV-2 has a zoonotic origin and that it is associated with respiratory and gastro-intestinal infections.

Because there is lack of a definite answer regarding whether the index case of COVID-19 was really due to zoonotic transmission, the current pandemic situation continues to generate misconceptions and misinformation in the public space [48]. One of the conspiracy theories describes SARS-CoV-2 as a biological weapon that was stealthily deployed during a covert spy operation between disputing nations. Other “infodemics” about COVID-19 include false claims of treatment breakthroughs, herbal remedies, biological mutation of the virus, and the right brand and proper usage of nose masks, among others [48].

## 3. Research and Development for Improved Response and Containment Operation Strategies

### 3.1. Importance of rapid, sensitive, and accurate point-of-care testing

According to the Chinese Center for Disease Control and Prevention (China CDC), clinical manifestations of COVID-19 are used in categorizing the patients into three disease groups- mild, severe, and critical. The mild COVID-19 manifestation category involves 81% of the patients. They often show no signs of pneumonia, or occasionally develop a mild symptom. However, the severe and critical cases present with a wide range of symptoms and pulmonary dysfunctions. Severe cases of COVID-19 occur only in 14% of patients, with the critical ones occurring only in 5% [49]. Asymptomatic SARS-CoV-2 carriers may be contagious [50], and a major barrier militating against rapid response and containment operations against COVID-19 is the failure to detect such virus shedders early enough to prevent the spread of the infection. Hence, urgent attention has been shifted to the development of newer, more rapid and accurate diagnostic tools to facilitate early contact tracing and rapid detection of COVID-19 cases, especially the asymptomatic individuals who may also shed the virus [42,51].

Several laboratory diagnostic methods – RT-PCR, serological tests such as ELISA among others have been developed and are currently in use. However, studies show that there is still a serious concern about the reliability of the major diagnostic tools for COVID-19 detection [52,53]. The reverse-transcriptase–polymerase-chain-reaction (RT-PCR) is considered a robust, specific, and reliable technique and has been well documented in the diagnoses of various viral infections such as ebolavirus disease, Zika, and influenza. Unfortunately, false-negative rates are now commonly reported for RT-PCR in COVID-19. Of note, Wikramaratna *et al*. [53] reported a high rate of 41% false-negative COVID-19 RT-PCR, indicating poor sensitivity akin to other laboratory tests with variable accuracy [54].

In a study that involved a systemic review of 957 patients, it was observed that false-negatives in RT-PCR assays could be as high as 29% [55]. A similar study involving more than a thousand test samples also assessed the accuracy level in RT-PCR assay and reported 38% median false-negative rate for the tests conducted on the 1^st^ day of symptom onset and 20% 3 days later [56]. Meanwhile, several diagnostic tests are available for COVID-19 patients, but until now there is no reference-standard test otherwise known as “gold standard” for SARS-CoV-2 tests.

In addition to molecular diagnostics, various rapid point-of-care kits for testing SARS-CoV-2 antibodies have been manufactured and marketed far and wide. These tests are basically qualitative and thus can only detect whether SARS-CoV-2 antibodies are present or not [57]. Besides, SARS-CoV-2 antibodies may be impacted by unavoidable cross-reactivity with other coronaviruses, especially the original SARS-CoV [52]. For these reasons, a great caution would be required in interpreting their results, especially to inform patients’ decisions.

Serology is fast evolving as a vital diagnostic tool for COVID-19 especially in spotting individuals with immunity against the SARS-CoV-2 infection, over time. Serological tests may be useful only for patients who present late, especially after the first 2 weeks following the onset of COVID-19 symptoms [52]. Even more concerning is the report that that the level of the earliest most sensitive antibody titer begins to rise only after the 2^nd^ week following the manifestation of symptoms [58]. In other words, serological test may not merit detection of early infection with SARS-CoV-2 especially in the presymptomatic, who are potential SARS-CoV-2 “silent spreaders.”

The common approaches of detecting asymptomatic patients are through massive screening and good follow-up. However, to wait for possible development of symptoms in both presymptomatic and “asymptomatic” individuals may not represent the best approach since these groups of patients could still fuel the contagion. Therefore, a rapid point-of-care test with improved sensitivity and specificity is the required tool. To achieve this, the Food and drug Administration (FDA) must lay down a “gold standard” test for all COVID-19 diagnostic tests as a reference point. Apart from the diagnostic methods, other challenges such as sampling and transportation problems are fast evolving and contribute to the unreliability of test outcomes. Among the sampling related factors are varying anatomic sites, improper labeling, and wrong virus cycle timing.

While inaccurate diagnostic results constitute a challenge to the containment of the pandemic, it is suggested that researchers should focus inwards on a range of methodological expertise, where accurate and early detection of SARS-CoV-2 could be made possible soon after contracting the disease. A previous report showed the potential use of respiratory virus testing panel (RVTP) for rapid diagnosis of COVID-19 [42], especially with possible rise in SARS-CoV-2 mutant strains. The development of RVTPs that are highly sensitive and specific to multi-strain SARS-CoV-2 targets, if commercially available, will no doubt aid in combating the transmission of SARS-CoV-2 more rapidly. It would also facilitate early confirmation of the suspected cases and identify the asymptomatic SARS-CoV-2 virus silent shedders. Since RVTPs may provide coverage for broader virus strains, its analytical sensitivity and specificity can be rapidly measured and identified. This would aid in shortening the turnaround diagnostic time and minimizing false results. It is also believed that RVTPs could circumvent the impacts of sampling and transportation errors, which may contribute to the bane of accurate testing. RVTPs may detect COVID-19 cases rapidly, especially if this is manufactured with a good reference guide to ensure high testing accuracy.

### 3.2. Antiviral drug therapies: Viral genomic organization, protein-protein interaction, and improved combination strategy against the emerging genetic variability

**Figure 2:**
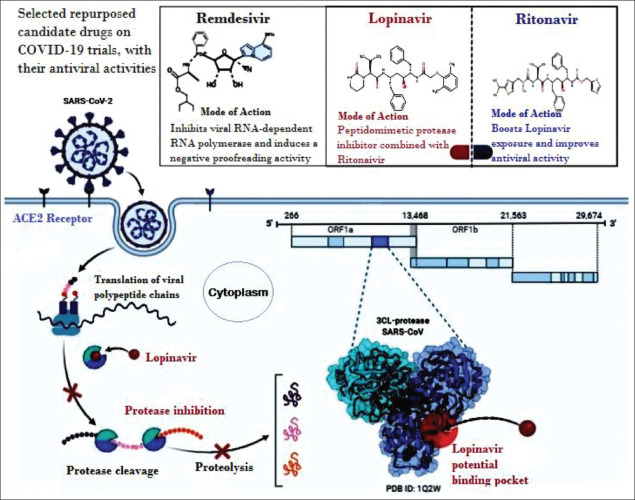
Selected potential repurposed candidate drugs for COVID-19. The potential drug targets and regions in the replication and enzymatic reaction phases are indicated.

The purpose of antiviral therapy is to lower viremia in patients in the acute phase of infection, to allow the body to recover, and to give the immune system time to mount up a specific response that will neutralize the pathogen and halt its continued spread. According to a recent report, there have been few completed clinical trials on the efficacy of antiviral therapy in the management of COVID-19 patients [59]. One of such clinical trials involved the addition of Lopinavir-Ritonavir to the standard treatment regimen of patients with severe COVID-19 (i.e., the use of antibiotics, invasive or non-invasive ventilation and extracorporeal membrane oxygenation (ECMO), and vasopressor). In effect, it did not improve clinical outcomes of 28-day mortalities and viral RNA load significantly [60]. It is, therefore, suggested that a combination of three antivirals: Lopinavir-Ritonavir and Remdesivir could provide a synergistic mechanism against SARS-CoV-2. However, such combination may induce adverse effects. Lopinavir and Ritonavir are protease inhibitors, previously used in the treatment of Acquired immunodeficiency syndrome (AIDS). They prevent viral replication by selectively binding to viral proteases, thus blocking the proteolytic cleavage of protein precursors required for viral replication [4]. Ritonavir inhibits the metabolism of Lopinavir and increases its plasma concentration [61] Table 1 and Figure 2.

Another antiviral agent Remdesivir, initially used in the therapeutic management of Ebola patients, is still undergoing wide-scale; multi-center clinical trials and the preliminary results suggest its efficacy in the treatment of patients with severe COVID-19 [62]. Remdesivir or GS-5734 is a broad-spectrum antiviral medication. It diffuses into cells where its active triphosphate metabolite GS-441524 interferes with the action of viral RNA-dependent RNA polymerase and evades proofreading by viral exoribonuclease (ExoN), leading to a reduction in viral RNA production [63]. Due to the emergence of varying mutants of SARS-CoV-2 and the genetic variability of individual patients, it may be important to consider synergistic effects or combinations of various potential repurposed drug candidates and their assessments when varying individual treatment options for management of COVID-19 (Figure 2).

In another recently concluded clinical trial, Veklury (Remdesivir) was superior to placebo in shortening the time to recovery in adults who were hospitalized with COVID-19 and had evidence of lower respiratory tract infection [64]. To help in accelerating the development of this promising drug, the Food and Drug Administration FDA through its coronavirus treatment acceleration program gave an approval for the use of Veklury (Remdesivir) in adults and pediatric patients [64]. Under a mechanism called emergency use authorization (EUA), Veklury (Remdesivir) can be used for treatment of suspected or laboratory confirmed COVID-19 in hospitalized pediatric patients weighing 3.5 kg to less than 40 kg or hospitalized pediatric patients less than 12 years of age weighing at least 3.5 kg [64]. Another agent; Bamlanivimab has also received EUA from the FDA as a monotherapy in adult and pediatric outpatients with mild-to-moderate COVID-19, and with positive results of direct SARS-CoV-2 viral testing who are 12 years of age and older weighing at least 40 kg, and who are at high risk for progressing to severe COVID-19 and/or hospitalization [65].

Moreover, other studies focusing on the structural genetics (structural and non-structural proteins SP/NSP) and mRNA biological mechanisms of SARS-COV-2 pathogenesis and immune evasion of the host cell will be beneficial in unlocking regions for drug targets, vaccine delivery and host immunological response. By addressing the therapeutic challenges based on the therapies that target the host-virus interface, where the emergence of mutational resistance is arguably less likely, could potentially present durable, broad-spectrum treatment modalities [66]. This could be achieved through systematic mapping of the interaction landscape between the SARS-CoV-2 proteins and human proteins, especially in emerging mutated strain of SARS-CoV-2.

Scientists have been able to unwind the SARS-CoV-2 host infectivity fuelled by enzymatic and antibody kinetics. SARS-CoV2 infection regulates host kinase signaling and virus kinase phosphorylation with its activity driven by host proteome [67]. The top kinase families predicted by sequence to regulate these sites included casein kinase II (CK2), cyclin-dependent kinase (CDK), and protein kinase C (PKC), among others, suggesting that these kinases may contribute to the regulation of viral replication. It is also observed that SARS-CoV-2 makes use of the host post-translational regulatory systems to promote rapid changes in cellular signaling [67]. Kinases represent ideal drug targets and the ongoing studies on the antiviral activity of 68 drugs and compounds approved by the FDA are aggressively pursued. Some of these agents are in clinical testing stage or under preclinical development for various diseases, including silmitasertib (CK2, phase 2), gilteritinib (AXL, FDA approved), ARRY-797 (p38, phase 2/3), MAPK13-IN-1 (p38, preclinical), SB203580 (p38, preclinical), ralimetinib (p38, phase 2), apilimod (PIKFYVE, phase 1), and dinaciclib (CDK, phase 3), among others [62]. Similarly, multiplexed enzyme-linked immunosorbent assay (ELISA) analysis of supernatants of cells from *in vitro* experiments demonstrated strong upregulation of inflammatory cytokines at the protein level. It is believed that these agents and diagnostic techniques could offer various potentials for viral inhibition; activation of host immunological response by T and B cells associated cellular proteins and the monitoring of disease duration.

### 3.3. Immunoglobulin therapy: Addressing the problem of quantity and specificity

The use of intravenous immunoglobulin (IVIg) therapy shows potential value as an effective adjunct measure in the treatment of several acute infections, including viral pneumonias such as SARS-CoV-2 [68]. According to a recent report, the commencement of IVIg <48 h of hospital admission to intensive care unit may prevent clinical demands such as the need for mechanical ventilation and the length of hospital stay. It also advances the early recovery of patients and improves considerable level of clinical efficacy [69].

However, a separate report implies that there is a limited benefit of IVIg therapy. It concluded that the treatment efficacy of IVIg would be better only if the immune IgG antibodies are pooled from the patients who recovered from COVID-19 in the same locality such as in the same city or the surrounding area. This can raise the possibility of neutralizing the virus [70]. The inherent limitations are that the harvested antibodies will not have widespread use and that the quantity that is available for use in the treatment of acute COVID-19 patients will depend on the number of recovered patients in each locality and the willingness of the individuals to donate immune sera. However, an alternative may lie in the use of cocktails of blocking monoclonal antibodies that specifically target and neutralize epitopes of predominant strains of SARS-CoV-2 circulating in wider geographical areas. Meanwhile, human monoclonal antibodies have been reported with the potential to block the binding of SARS-CoV-2 spike protein to angiotensin-converting enzyme 2 receptor sites of host cells [71]. A monoclonal antibody targeting the spike protein S1 of SARS-CoV-2, made from immunized transgenic mice expressing human Immunoglobulin (Ig) was reported to neutralize both SARS-CoV-2 and SARS-CoV infection [3].

At present, various Igs are showing fruitful experimental outcomes as a promising class of drugs, it is believed that these proteins can, therefore, be produced *ad infinitum* in cell culture vats using hybridoma technology. Hybridoma technology could circumvent the need to pool intravenously or recruit patients who recovered from the disease once the genetic variants are fully identified. This may remove the barrier of the limited amount of harvested sera, and obviate the problem of the evolving constant mutation by the virus, using specific monoclonal antibodies through this technology. In this process, an antigen (or its epitope) is injected into a mammal to stimulate its immune system’s B-cells and produces neutralizing antibodies specific to the antigen. Furthermore, the antibody-producing B-cells are harvested and fused with a myeloma (immortal B cell cancer cells) *in vitro* to produce a hybrid cell line (a hybridoma), which retains the antibody-producing ability of the B-cell and the reproducibility of the myeloma.

A collection of various SARS-CoV-2 hybridomas from varying genetic mutants and preparation of a range of serotypes from immunized cells, followed with a proper characterization and purification processes could be a novel idea to produce cocktails of SARS-CoV-2 blocking monoclonal antibodies. Likewise, the use of Ig combination specific to different antigenic sites from different strains may limit the problems of treatment resistance occasioned by genetic variants and immune escape and thus offer a useful treatment option.

### 3.4. COVID-19 vaccines

#### 3.4.1. Addressing the immunoreaction cum evolution of SARS-CoV-2 mutant strains

The SARS-CoV-2 has demonstrated a remarkable ability to mutate. There has been an identified unexpected relationship between the SARS-CoV-2 mutation densities and viral transmission dynamics at different levels among human populations. A total of 14 new strains of the virus apart from the one originally identified in Wuhan have been characterized, and each has reportedly fueled regional epidemics such as those witnessed in Asia, Europe, North America, and Latin America [72]. Spike mutation pipeline reveals the emergence of a more transmissible form of SARS-CoV-2. A pathogen that mutates rapidly will, each time, present a new epitope, antigenic determinant or antigen molecule to which an antibody attaches, to the immune system. Meanwhile, the immune system is expected to respond by producing newer, specific antibodies that will bind to and neutralize the new epitope. It is, therefore, imperative to state that not all antibodies produced against epitopes of different strains of a pathogen are cross-protective. It is perhaps why an increasing number of COVID-19 patients are re-testing positive after recovery, although still unclear at this time if this is a result of the reactivation of the virus in the patients as opposed to re-infection [73,74].

It is important at this juncture, to add that evolving mutational significance in SARS-COV-2 termed synonymous and non-synonymous mutations is imminent. About this, mutational changes have been identified from COVID-19 cases in the highest hit countries including the United State of America (USA) and United Kingdom (UK) [75]. Studies have shown that SARS-COV-2 14408 C>T and 23403 A>G mutations are associated with an increase in mutation density over time [75]. The report further shows that there exists a strong positive correlation between non-synonymous mutational densities and time with 14408 C>T/23403 A>G genotypes of SARS-CoV-2 in both the UK and US. These changes were identified among the “wild type” and “mutant” strains of SARS-CoV-2 [75]. Understanding the biological disparities between the “mutant” and “wild type” strains of SARS-CoV-2 would assist in the make-up of the vaccine ingredients and measurement of the mutational densities in synonymous and non-synonymous mutant strains.

#### 3.4.2. Authorization of emergency COVID-19 vaccines with many unanswered questions

There is good news about the emergency use authorizations (EUAs) of COVID-19 vaccines sponsored by the Pfizer/BioNTech, Moderna, and AztraZeneca. The U.S.- FDA has recently approved some new recombinant protein-based, non-replicating subunit RNA vaccines against COVID-19. So far, the Phase 3 clinical trial results show that these vaccines can be safe and effective for use. However, this is EUA and not a full approval. According to the FDA [76], EUA is given when adequate and approved alternatives are unavailable; it does not mean these vaccines are completely risk-free.

It is believed that the current demanding COVID-19 situations in places like the United States and some parts of the Europe had called for the emergency authorization. Many questions in association with these vaccines are yet unanswered. It is reasonable to accept that there are potential risks and fears of unknown regarding these new vaccines. But making these vaccines hastily available faster than the usual process should be less contemplated against the risk of infection with COVID-19 itself. However, it is believed that when productions of these vaccines increase and become more readily available and when many populations are vaccinated, there could be possible emergence of unknowns at any time. These unknowns will prompt scientists to continue to ask questions. It is important that some of the puzzles about emergency use of COVID-19 vaccines be brought to the limelight of discussion, especially now that there seem to be inadequate time to follow-up with the trial participants before the emergency approval. Now that vaccination already kick started, how long will the vaccine protection last? From participant recruitment to FDA-emergency approval, the process of clinical trials of these vaccines took less than 6 months. Therefore, it is believed that there were no adequate follow-ups to answer this question. Similarly, there is no current available data to understand whether the vaccines will require booster shots and the perfect time these might be expected.

Besides, the main goal of COVID-19 vaccines is to prevent personal infection. Then, will the vaccinated individuals still prevent the spread of SARS-CoV-2 virus to non-vaccinated individuals? Even with the high level of assurance being provided by the vaccine companies regarding their efficacies (especially the mRNA vaccines) against all forms of SARS-CoV-2 mutant strains, it remains to be acknowledged whether the vaccinated individuals can still spread the infection or not. While in the present situations, it may be virtually impossible to vaccinate everyone due to the logistic and economic reasons, this question demands immediate answer in containment operations.

In addition, the available vaccines data did not provide information regarding effectiveness of the vaccines in specific groups of people such as asymptomatics, immunocompromised, previously infected individuals, pediatrics, pregnant, and breastfeeding women, among many others. It is important to note that “efficacy” in clinical trials is not tantamount to “effectiveness” in clinical applications to all population groups. As at the time of writing this report, none of the clinical trials have answered questions regarding these specific individuals, yet they were so listed in their exclusion criteria in clinical trial statement [77].

Furthermore, with the euphoria of arrival of the first COVID-19 vaccines, the virus seems to continue “re-strategizing” its battle with the emergence of new mutant strains, rapidly spreading, and affecting younger populations [78]. Now that we have emergency vaccine available, can the people infected with the new variants (such as UK- 20B/501Y.V1 and South African-20C/501Y.V2 strains) be protected? The answers we seek to these questions would be very germane to human safety and in addressing the global threats of COVID-19 disease that has affected socio-economic well-being in many parts of the world.

## 4. Conclusions

Detailed information about the biology of SARS-CoV-2 is currently unknown. However, more information on the ecology of the virus is now in the limelight. The problem of SARS-CoV-2 rapid mutations is an emerging area of concern demanding urgent consideration in clinical trials and patient management. Vaccines usually become ineffective when a virus mutates. Likewise, drug resistance ensues when the genetic component of a virus is unstable. Ongoing clinical trials and current patient management on COVID-19 would achieve landmark success if the following are considered: (i) Improved rapid, sensitive and accurate diagnostics and development of a standard test, (ii) addressing the constant evolution of SARS-CoV-2 mutant strains, (iii) addressing the problem of immunoglobulin specificity prospectively, and (iv) Improving antiviral combination strategy against emerging genetic variability.
